# Supplementary material for: Impact of an In-Hospital Endocarditis Team and a State-Wide Endocarditis Network on Perioperative Outcomes
Source: J Clin Med. 2021 Oct 15;10(20):4734. doi: 10.3390/jcm10204734 (PMC8541635; doi:10.3390/jcm10204734)
Supplement: Supplementary file 1 [file jcm-10-04734-s001.zip › jcm-1408260-SI.pdf]

Supplementary materials to:

Impact of an In-hospital Endocarditis Team and a State-wide Endocarditis Network on Perioperative Outcomes

Content

Supplementary Table S1 Microbiological findings of IE patients operated in the 1st era (2007-2014) and in the 2nd era (2015-2018) 3

Supplementary Table S2 Risk factors for in-hospital mortality by multivariate logistic regression analysis 4

Supplementary Table S3 Risk factors for post-operative stroke by multivariate logistic regression analysis 5

Supplementary Table S4: Pre-operative endocarditis-related complications as well as post-operative outcomes for the patients treated between 2011 and 2014 compared to those treated between 2015 and 2018. 6

Figure legends 7

- Supplementary Figure S1 7
- Supplementary Figure S2 7
- Supplementary Figure S3 7

Supplementary Table S1 Microbiological findings of IE patients operated in the 1st era (2007-2014) and in the 2nd era (2015-2018)

	First era 2007-2014	Second era 2015-2018	p-value
No findings	60 (15)	12 (6)	0.001
Staphylococcus aureus	102 (25)	73 (34)	0.026
coagulase-negative Staphylococcus	60 (15)	43 (20)	0.113
Enterococci	63 (15)	31 (14)	0.814
Streptococci	100 (24)	32 (15)	0.005
HACEK	2 (1)	5 (2)	0.052
other	22 (5)	21 (10)	0.047
multiple pathogens	31 (8)	11 (5)	0.139

Data are given as numbers (%). HACEK: Haemophilus, Cardiobacterium, Eikenella, Kingella.

Supplementary Table S2 Risk factors for in-hospital mortality by multivariate logistic regression analysis

	Adjusted OR	95% CI	*P* Value
No ET recommendations	2.12	1.27-3.53	**0.004**
Age	1.06	1.03-1.08	<**0.001**
S. aureus IE	2.61	1.58-4.32	<**0.001**
Pre-operative Bilirubin	1.03	1.01-1.04	<**0.001**
LVEF	0.98	0.95-0.99	**0.018**
Multiple-valves IE	1.89	1.17-3.04	**0.008**
Antiplatelet therapy	0.62	0.61-0.37	0.056
BMI	1.05	0.99-1.10	0.072
Pre-operative hemodialysis dependency	1.70	0.96-2.97	0.062

CI: confidence interval; BMI: body mass index; ET: endocarditis team; IE: infective endocarditis; LVEF: left ventricular ejection fraction OR: odds ratio

Supplementary Table S3 Risk factors for post-operative stroke by multivariate logistic regression analysis

	Adjusted OR	95% CI	*P* Value
Pre-operative artificial ventilation	2.00	1.01-3.92	0.045
No ET Recommendations	2.23	1.12-4.39	0.021
Mitral valve IE	2.06	1.08-3.92	0.028
S. aureus IE	1.87	0.98-3.53	0.055
Cardiac abscess	1.94	1.04-3.63	0.037

CI: confidence interval; ET: endocarditis team; IE: infective endocarditis; OR: odds ratio.

Supplementary Table S4: Pre-operative endocarditis-related complications as well as post-operative outcomes for the patients treated between 2011 and 2014 compared to those treated between 2015 and 2018.

	**2011-2014** (n=234)	**2015-18** (n=221)	**p-value**
Time form symptom to referral	14 (6-33)	7 (2-19)	<0.001
Time form symptom to surgery	18 (10-38)	9 (4-21)	<0.001
Pre-operative NYHA ≥ III	157 (67)	98 (45)	<0.001
Pre-operative acute renal failure	28 (12)	27 (8)	0.157
Cardiac Abscess	76 (33)	54 (24)	0.062
Pre-operative stroke	60 (26)	30 (14)	0.001
Post-operative stroke	30 (13)	10 (5)	0.002
Post-operative hemodialysis	45 (19)	48 (22)	0.561
Length of ICU stay	5 (2-13)	7 (4-14)	0.992
Length of hospital stay	18 (12-34)	20 (14-28)	0.580
In-hospital mortality	63 (27)	54 (24)	0.316
One-year mortality	93 (40)	79 (36)	0.386
Re-endocarditis	23 (10)	21(10)	1.00

Data are given as median (interquartile range, 25th–75th percentile) or n (%).

ICU: intensive care unit; NYHA: New York Heart Association.

Figure legends

Supplementary Figure S1: The percentage of patients diagnosed in our hospital (green) or referred from other hospitals (blue) during the 2007-2014 compared to during 2015-2018.

Supplementary Figure S2: Kaplan-Meier survival estimates of patients operated during the 1st era (2007-2014), blue line) compared to those operated in the 2nd era (2015-2018, red line).

Supplementary Figure S3: Kaplan-Meier survival estimates of patients treated according to recommendations of the endocarditis-team (red line) compared to those not treated according to the recommendations of the endocarditis-team (blue line)

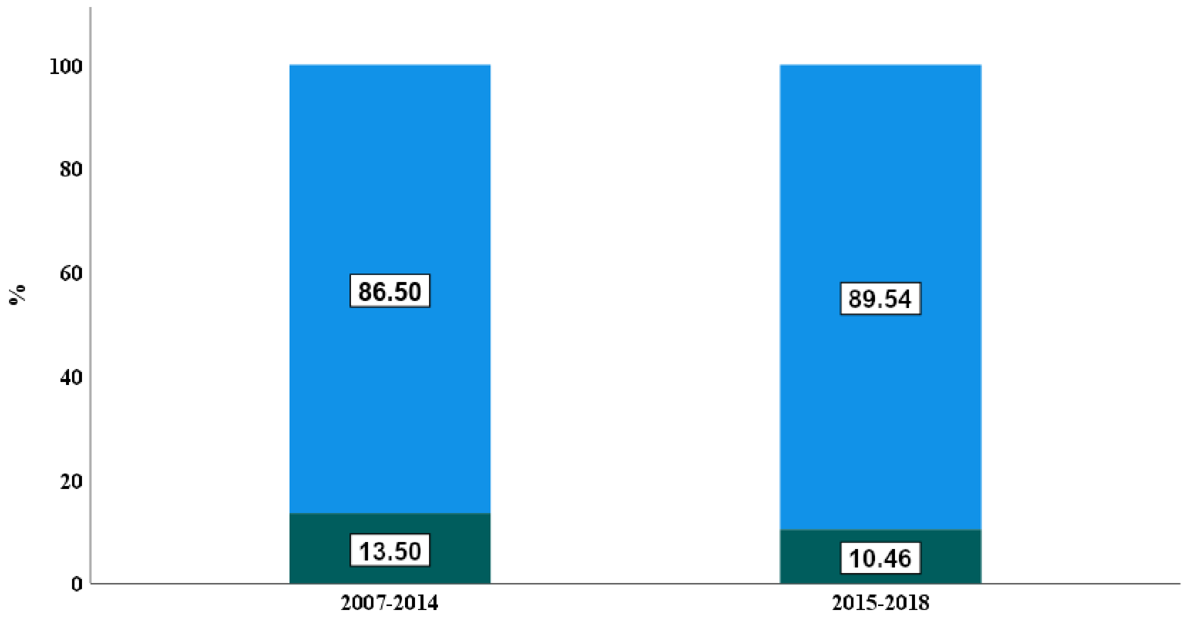

Supplementary Figure S1: The percentage of patients diagnosed in our hospital (green) or referred from other hospitals (blue) during the 2007-2014 compared to during 2015-2018.

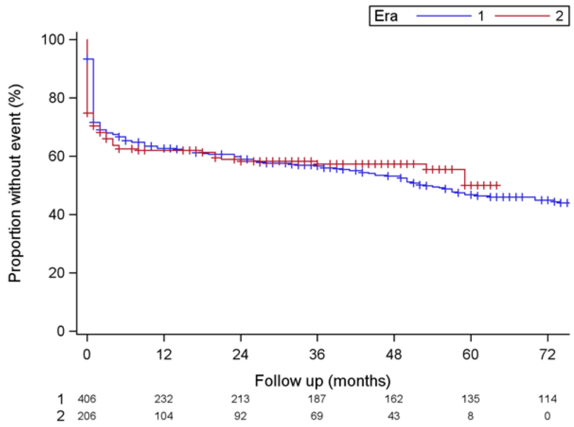

Supplementary Figure S2: Kaplan-Meier survival estimates of patients operated during the 1st era (2007-2014), blue line) compared to those operated in the 2nd era (2015-2018, red line).

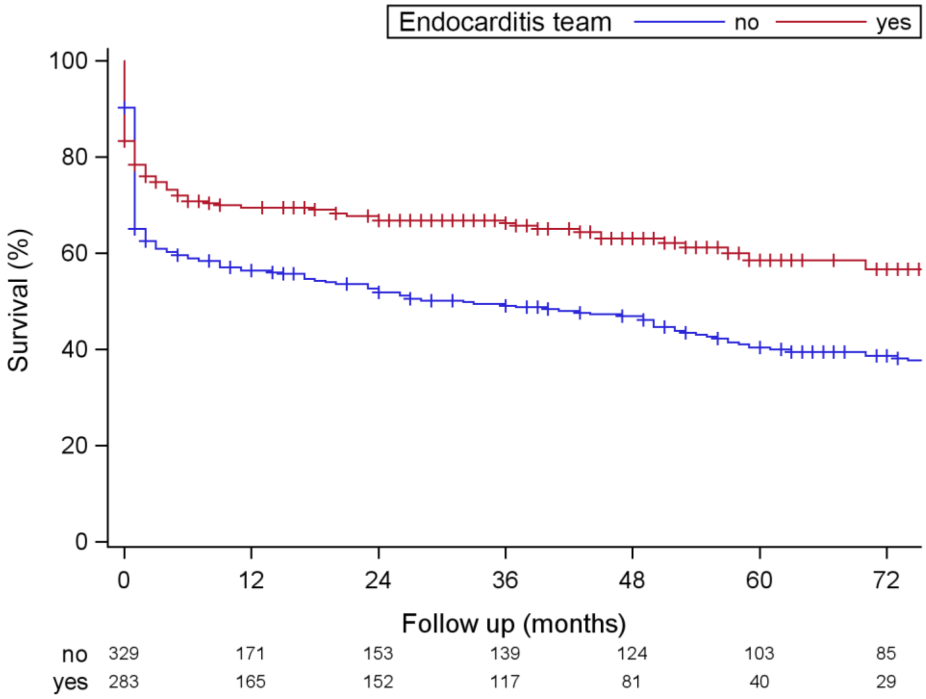

Supplementary Figure S3: Kaplan-Meier survival estimates of patients treated according to recommendations of the endocarditis-team (red line) compared to those not treated according to the recommendations of the endocarditis-team (blue line)
